# Inequalities in Retirement Life Span in the United States

**DOI:** 10.1093/geronb/gbac180

**Published:** 2022-11-17

**Authors:** Jiaxin Shi, Christian Dudel, Christiaan Monden, Alyson van Raalte

**Affiliations:** Max Planck Institute for Demographic Research, Rostock, Germany; Leverhulme Centre for Demographic Science, Department of Sociology, University of Oxford, Oxford, United Kingdom; Federal Institute for Population Research, Wiesbaden, Germany; Max Planck Institute for Demographic Research, Rostock, Germany; Federal Institute for Population Research, Wiesbaden, Germany; Leverhulme Centre for Demographic Science, Department of Sociology, University of Oxford, Oxford, United Kingdom; Nuffield College, University of Oxford, Oxford, United Kingdom; Max Planck Institute for Demographic Research, Rostock, Germany

**Keywords:** Education, Gender, Inequality, Retirement, Work-related issues

## Abstract

**Objectives:**

The length of retirement life may be highly unequal due to persistent and significant discrepancies in old-age mortality. This study assesses gender and educational differences in the average retirement life span and the variation in retirement life span, taking into account individual labor force exit and reentry dynamics.

**Methods:**

We used longitudinal data from the Health and Retirement Study from 1996 to 2016, focusing on respondents aged 50 and older (*N* = 32,228). Multistate life tables were estimated using discrete-time event history models. The average retirement life span, as well as absolute and relative variation in retirement life span, were calculated analytically.

**Results:**

Among women, we found a persistent educational gradient in average retirement life span over the whole period studied; among men, the relationship between education and retirement expectancy differed across periods. Women and the lower-educated had higher absolute variation in retirement life span than men and the higher-educated—yet these relationships were reversed when examined by relative variation.

**Discussion:**

Our multistate approach provides an accurate and comprehensive picture of the retirement life span of older Americans over the past two decades. Such findings should be considered in high-level discussions on Social Security. Potential reforms such as raising the eligibility age or cutting benefits may have unexpected implications for different social groups due to their differential effects on retirement initiation and reentry dynamics.

Retirement is a stage in life where individuals have more control over both the pace and the content of their lives (Ghilarducci & Webb, 2018). Thus, living a long life in retirement is a desirable goal of many people. As mortality reductions at ages 65 and older have been the main driver of life expectancy (LE) gains since the mid-20th century (Rau et al., 2018), people are expected to live longer in retirement now than in the past.

The lifetime spent in retirement is determined by more than just the timing of death (Crimmins et al., 2018; Kibele et al., 2013). Two other factors shape retirement life span: first, the variation in the time of withdrawal from the labor force (Bernheim, 1989); second, labor force reentry after a phase of retirement (Cahill et al., 2015). Over the period 1992–2004, half of the U.S. men and women had exited the labor force by ages 63 and 61, respectively, and one third of the population returned to work after an initial exit from the labor force (Warner et al., 2010). The actual time spent in retirement is far from self-evident due to the uncertainty in these three factors.

The age at death, the age at initial retirement, and the usage of labor force reentry are known to vary across individuals with different characteristics. First, women tend to live longer and retire earlier than men (Hanson & Wapner, 1994). Second, education not only affects life span but also retirement patterns (Hayward et al., 1994). Because of these differences, separate analyses of retirement life span by gender and education are required.

In this study, we investigated gender and educational differences in retirement life span using data on synthetic cohorts from the Health and Retirement Study (HRS) spanning over two decades (1992–2016). Retirement life span is the variable defined as the time spent in retirement beyond the age of 50 years. Discrete-time event history analysis and multistate life tables were used to model career transitions. We focused on several dimensions of inequalities, including the *average* lifetime spent in retirement, or “retirement expectancy,” and the *variation* in retirement life span, or “retirement lifespan variation,” and examined how they vary by gender and education.

Within-group variation has largely been overlooked in the context of retirement life span, although it has been actively studied in mortality research under the label of *life span variation*. Complementary to LE, variation metrics have gained increasing attention among researchers, particularly for studying educational differences in mortality (Brown et al., 2012; Sasson, 2016; van Raalte et al., 2011, 2014, 2018). Variation metrics capture how much individuals of the same group differ in their life spans, that is, the within-group heterogeneity in survival.

Similarly, variation metrics can be used to capture within-group inequalities in retirement life span. For policymakers, if ensuring fairness in the length of life in retirement is a desirable goal, the within-group variation is another dimension of policy fairness in addition to the average retirement life span. Monitoring variation is also useful for policies that aim to distribute resources for the retired population because knowing the average needs is insufficient. Furthermore, variation indicates the level of uncertainty for individuals from a probabilistic perspective (Courgeau, 2012), potentially affecting individual decisions in saving, consumption, and investment.

We contribute to the literature in three ways. First, inequalities in retirement expectancies (RE) have rarely been directly measured, despite mounting concerns that pension reforms could exacerbate current inequalities in years of retirement (Beach & Bedell, 2019). Second, in contrast to earlier literature that focuses on LE at Social Security full retirement age, we measure retirement life span more accurately by considering all transitions: retiring, returning-to-work, and dying. Third, we are the first to apply the concept of variation to the study of inequalities in retirement length, bringing together two previously independent strands of literature: research on life span variation and research on retirement transitions. Our research is fully reproducible; we provide *R* codes (Shi et al., 2022), making it straightforward to apply our concepts to other data.

## Background

### Retirement Onset and Labor Force Reentry in the United States

Inequalities in the time spent in retirement have often been gauged by differences in remaining LE at age 65 (LE_65_; Kibele et al., 2013; Majer et al., 2011). Yet the actual age of initial retirement varies across individuals, especially in the United States. The official Social Security full retirement age in the United States increased from 65 to 66 years for cohorts born in 1943–1954, and it will increase further for cohorts born in 1955 and later (Behagel & Blau, 2012). The youngest age at which Social Security pension benefits can be claimed is 62 years. The actual ages at which individuals retire are often below the upper threshold, and sometimes even below the first claiming threshold (Warner et al., 2010).

On average, women retire earlier than men; this observation is consistent across time, yet gender differences in retirement age have narrowed more recently (Quinn et al., 2011). Gender differences in retirement patterns can be partly explained by family circumstances, as women more often quit paid work to take on unpaid domestic and care work (Fisher et al., 2016).

Research across the world has consistently found an effect of education on retirement timing. The self-expectation of working beyond the age of 65 was higher among higher-educated people than among the lower-educated (Mermin et al., 2007; Szinovacz et al., 2014). This matches the observed patterns where lower education is associated with earlier retirement, while higher education is associated with later retirement (Damman et al., 2011; Zickar, 2013). In the United States, Venti and Wise (2015) found that lower-educated people claimed Social Security benefits earlier than higher-educated people. One explanation is that higher income and better work conditions attract higher-educated people to work longer (Potočnik et al., 2009). Ill health has also been found to contribute to the association between low education and early retirement (Jung et al., 2020; Lawless et al., 2015). It is also possible that higher-educated people delay their retirement to compensate for later career onset and to recoup their earlier investment in education (Fisher et al., 2016).

The association between education and retirement may vary over time. During the 2008–2009 Great Recession, the probability of being retired at age 65 increased for both men and women, as older workers were pushed out of the labor market (Dudel & Myrskylä, 2017), but the impact of the recession varied greatly by education, affecting those with less education disproportionately (Hale et al., 2021).

Labor force reentry after initial retirement is another key factor shaping retirement life span. Reentry is a common phenomenon in the United States (Cahill et al., 2015; Warner et al., 2010). Skoog and Ciecka (2010) showed that work history predicted one’s propensity to return to work. A 65-year-old man who was still active in the labor market was unlikely to reenter after the initial exit from the labor force, whereas a man who was inactive at age 65 was much more likely to reenter after his initial retirement (Skoog & Ciecka, 2010). As gender and education are associated with work history, they may influence the probability of reentering the labor force. In general, women were less likely to engage in postretirement work than men (Maestas, 2010; Pleau, 2010). Hayward et al. (1994) showed that, among all the retirees, lower-educated men were more likely to take part-time jobs after initial retirement than higher-educated men.

### U.S. Older-Adult Mortality: Life Expectancy and Life Span Variation

In addition to the timing of (un)retirement, mortality is another key component determining the retirement life span. After a long period of gradual increase, LE in the United States plateaued in recent years (Dyer, 2018), prior to the coronavirus disease 2019 (Covid-19) pandemic. This trend has been explained by increasing overdose mortality over younger-adult ages and slow declines in mortality related to circulatory diseases at middle to older ages (Mehta et al., 2020). The worrisome trend of LE in the United States is also partly attributable to divergent developments in mortality across socioeconomic groups, as studies have found that individuals with lower education and income have experienced declining LE since 1990 (Chetty et al., 2016; Meara et al., 2008; Sasson, 2016; Sasson & Hayward, 2019).

In population health research, variation metrics have been increasingly used to examine group differences in within-group life span inequality. Researchers find that men and individuals with lower socioeconomic status (SES) tend to have larger life-span variation in the United States in addition to their shorter LE. This occurs when looking at the variation over the full range of adult ages (Sasson, 2016; Sasson & Hayward, 2019). It is also the case when comparing expectancies and variation in ages at death above the mode (Brown et al., 2012). In other words, the health of men and lower SES groups are more heterogeneous than it is for women and higher SES groups. The life-span variation from a fixed old-age threshold onward has been found to follow an upward trend for the entire population (Engelman et al, 2014; Myers & Manton, 1984). For SES-specific trends in old-age life-span variation above age 65, findings are mixed across countries, gender, and education (Zarulli et al., 2012).

### Hypotheses

Men and more educated individuals tend to retire later, and men and lower-educated individuals are more likely to return to work. Therefore, we expect that men have shorter retirement life spans than women, whereas the relationship between education and retirement life span is unclear. On the one hand, more educated people tend to live longer, which can lead to longer retirement life spans. On the other hand, they are more likely to postpone their retirement (Venti & Wise, 2015), thus reducing their retirement life spans. As divergence in adult life-span variation has been driven by diverging mortality in working ages (Sasson, 2016), it is less clear whether retirement life-span variation has diverged across educational groups, particularly given the unknown labor force dynamics at older ages and the potential differences by gender and education.

## Method

### Data

We used the Health and Retirement Study (HRS), a biennial cohort-based panel since 1992 that contains a representative sample of noninstitutionalized individuals aged 50 and older in the United States. We created yearly work trajectories using 1996–2016 waves. Analyses were restricted to individuals aged between 50 and 100 at the time of the interview, including respondents and their age-eligible spouses (*N* = 32,228).

### Outcome Variable

**Figure 1.**
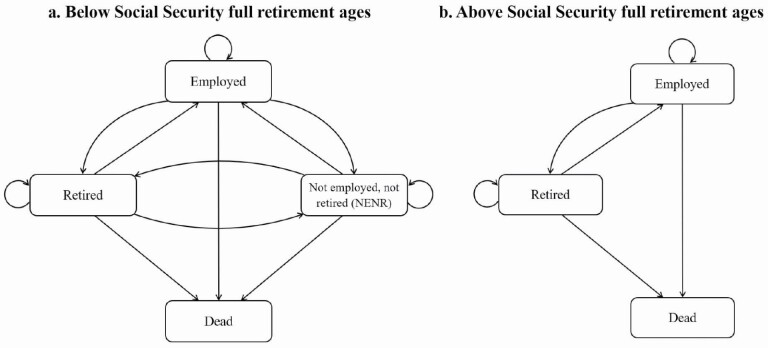
Transitions between states between age 50 and cohort-specific Social Security full retirement ages (panel a) and above Social Security full retirement ages (panel b). Source: Authors’ own. *Note*: When below cohort-specific Social Security full retirement ages, individuals who are unemployed or out of the labor force but do not identify themselves as retired are classified as “not employed, not retired (NENR).”

We classify individuals into three mutually exclusive states below the Social Security full retirement age: (a) “employed,” (b) “retired,” and (c) “out of the labor force (but not retired) or unemployed” (i.e., not employed, not retired [NENR]), using self-reported information (Dudel & Myrskylä, 2017). Figure 1 presents possible transitions between states. “Employed” includes self-employed individuals and those who are either working or on temporary leave such as sick leave or holiday. The classification follows this procedure: first, if individuals report themselves as employed, they are classified as “employed.” Second, for individuals who report themselves as not employed, they are classified as “retired” only if they report themselves as retired. Third, those who are left from the first two procedures are classified to the state NENR. For ages above cohort-specific Social Security full retirement ages, people are either “retired” or “employed”. Those who report themselves as not employed and not retired are automatically classified as retired.

### Predictors

We measure education by the highest degree obtained; it has three levels: below high school diploma; a high school diploma or a GED; and a college or university degree (see Author Note 1). Other key predictors include gender, the employment state in the preceding year, and period dummy variables for 2000–2003, 2004–2007, 2008–2011, and 2012–2015 (with the period 1996–1999 as the reference group). Age is included using a smoothing spline (Debón et al., 2006; Yee & Wild, 1996), together with three dummy variables for *Age 62–64, Age 65*, and *Age 66* capturing institutional retirement entry, and one dummy variable *Age 67+* to capture older-age retirement entry.

### Statistical Analyses

Ideally, we would be interested to observe complete later-life work-retirement histories. However, complete cohort data where everyone has died is rarely available. A solution is to use the *synthetic cohort approach*—a method that is commonly used by demographers. In this approach, the conditions of a given period, such as a year or a few years are assumed to prevail throughout the lives of members of an artificial cohort. One advantage of this approach is that it reflects temporal changes in mortality and retirement behavior, and provides provisional answers to timely important questions. This synthetic cohort approach has been used by many previous studies on old-age labor market activities and health transitions (Leinonen et al., 2018; Skoog & Ciecka, 2010; Warner et al., 2010; West & Lynch, 2020). Our synthetic cohorts each correspond to one of the five periods mentioned earlier.

First, we use multinomial logistic regression models to estimate the probabilities of transitions between states. Besides the predictors mentioned earlier, the interaction terms between education and period and between education and age dummies are also included. Survey weights are used. All models are estimated separately for men and women. The survival probabilities resulting from these models are adjusted such that they match the survival probabilities provided by the Human Mortality Database (HMD, 2022).

Subsequently, for each period–gender–education combination, we use the predicted year-to-year transition probabilities to analytically derive (a) probabilities of dying without retiring and (b) distributions of state occupation time (Dudel, 2021). This assumes that transitions between states follow the Markovian processes, that is, transition probability from time (age) *t* to time *t* + 1 only depends on the state at time *t*, not prior transition histories.

Retirement expectancy (RE) is calculated as the average of the distribution of time spent in the state “retired.” We use both absolute and relative inequality measures for retirement life-span variation. Absolute inequality is translation-invariant (i.e., inequality remains invariant when all individuals gain the same number of years of life in retirement), whereas relative inequality is scale-invariant (i.e., inequality remains invariant when all individuals gain the same proportional change in years of life in retirement). Absolute and relative measures provide complementary perspectives on inequality and may sometimes lead to different results. We use the average interindividual difference (AID) to measure absolute retirement life-span variation. The AID can be interpreted as the average difference in retirement life span between any two random individuals. It is calculated as:


$$\begin{array}{l} AID=\frac{\overset{n}{\underset{i=1}{\sum }}\overset{n}{\underset{j=1}{\sum }}\left|x_{i}-x_{j}\right|}{2n^{2}} \end{array}$$

where $x_{i}$ and $x_{j}$ are the retirement life spans for individuals i and j, and n is the total number of individuals. We use the Gini coefficient (G), a commonly used inequality measure in the literature, for relative inequality. It is calculated as:


$$\begin{array}{l} G=\frac{AID}{RE} \end{array}$$

We use the bootstrap method to calculate 95% confidence intervals.

## Results

**Figure 2.**
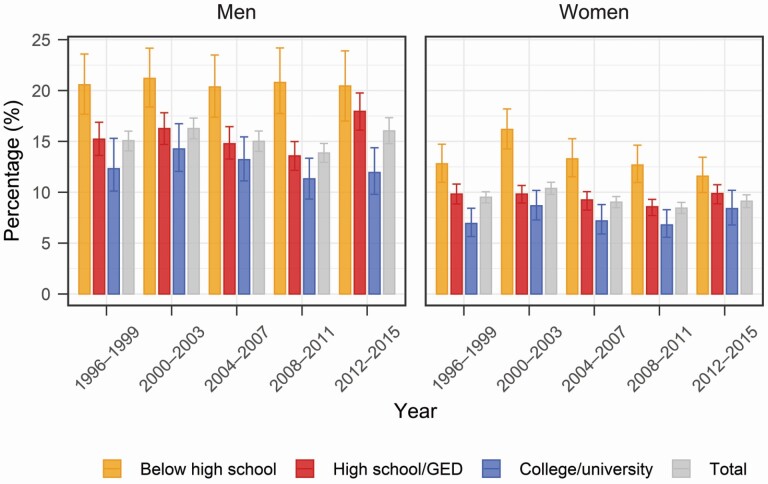
Percentage of individuals not surviving to retirement. Source: Authors' calculation based on the Health and Retirement Study, 1996–2016. *Note*: Error bars show 95% bootstrap confidence intervals.

Not all adults reach retirement. Figure 2 shows the percentages of people who die without retiring conditional upon survival to and not being retired at age 50. Men were more likely to die before retirement than women in all periods for all education subgroups. On average, the probability of dying without ever retiring was around 15% for men and below 10% for women. This is partly explained by men’s higher mortality and higher employment rates. Indeed, men were more likely to be employed at age 50 than women (see Author Note 2), and these gender differences in employment rates persist to older ages.

Higher education was associated with a lower percentage of dying before retirement for both men and women, despite higher labor force participation rates among the higher-educated. In 1996–2015, the average difference in the percentage of not surviving to retirement between the lowest and highest education groups was 5.7% for women and 8.1% for men. Educational percentage-point differences in preretirement mortality were stable for men but decreased from 7.6% in 2000–2003 to 3.2% in 2012–2015 for women.

For those *who were not retired at age 50 and survived to retirement*, Table 1 shows their mean initial retirement age, LE, RE, expected years in labor force reentry at the initial retirement age, and the percentage of LE in reemployment (see Author Note 3). Among the lowest educated, women retired later than men; whereas the gender difference is reversed in the two higher education groups. Overall, for both genders, the initial retirement age was positively associated with education. For women, there were no educational differences in reemployment expressed either as expected years or a proportion of LE at initial retirement; whereas for men, a positive educational gradient was found in both.

**Table 1.** Initial Retirement Age, Life Expectancy (LE), Retirement Expectancy (RE), Expected Years in Reemployment at Initial Retirement, and Percentage of LE in Reemployment

	Men					Women				
	1996–1999	2000–2003	2004–2007	2008–2011	2012–2015	1996–1999	2000–2003	2004–2007	2008–2011	2012–2015
Initial retirement age										
Total	63.5	64.5	64.5	64.2	65.9	63.8	64.9	64.5	64.4	65.4
Below high school	63.5	63.6	63.7	63.2	64.5	63.9	64.6	64.4	64.3	65.0
High school/GED	62.7	64.0	63.9	63.8	65.4	64.0	64.8	64.4	64.1	65.0
College/university	64.6	65.9	66.2	66.1	67.9	63.3	66.1	65.2	65.4	67.0
LE at initial retirement										
Total	16.2	15.9	16.7	17.7	16.2	19.7	18.9	19.7	20.4	19.8
Below high school	14.3	14.4	14.9	15.0	15.2	18.4	16.4	17.9	18.3	19.2
High school/GED	16.3	15.8	16.8	17.7	15.2	19.5	19.4	20.1	20.9	20.0
College/university	17.1	16.4	16.9	18.4	17.7	21.3	18.9	20.8	21.4	19.9
RE at initial retirement										
Total	14.4	13.9	14.6	15.6	14.0	17.7	16.8	17.7	18.4	17.7
Below high school	12.7	12.8	13.3	13.5	13.4	16.6	14.6	15.9	16.3	17.2
High school/GED	14.5	13.8	14.8	15.5	12.9	17.4	17.2	17.9	18.8	17.8
College/university	15.0	14.1	14.5	15.8	15.1	19.5	16.7	18.7	19.2	17.6
Expected years in reemployment										
Total	1.8	2.0	2.1	2.1	2.2	2.0	2.1	2.0	2.0	2.1
Below high school	1.6	1.6	1.7	1.6	1.8	1.8	1.8	1.9	2.0	2.0
High school/GED	1.8	2.0	2.0	2.2	2.2	2.1	2.2	2.2	2.2	2.2
College/university	2.1	2.3	2.4	2.6	2.6	1.8	2.1	2.1	2.2	2.3
LE% in reemployment										
Total	11.2	12.6	12.3	12.1	13.7	9.9	11.0	10.2	9.9	10.6
Below high school	11.1	11.1	11.1	10.4	11.9	9.7	11.0	10.8	10.7	10.4
High school/GED	10.9	12.7	12.1	12.3	14.8	10.8	11.3	10.8	10.4	11.1
College/university	12.4	14.2	14.2	13.9	14.9	8.4	11.4	10.2	10.2	11.8

*Notes*: Source: Authors’ calculation based on the Health and Retirement Study, 1996–2016.

**Figure 3.**
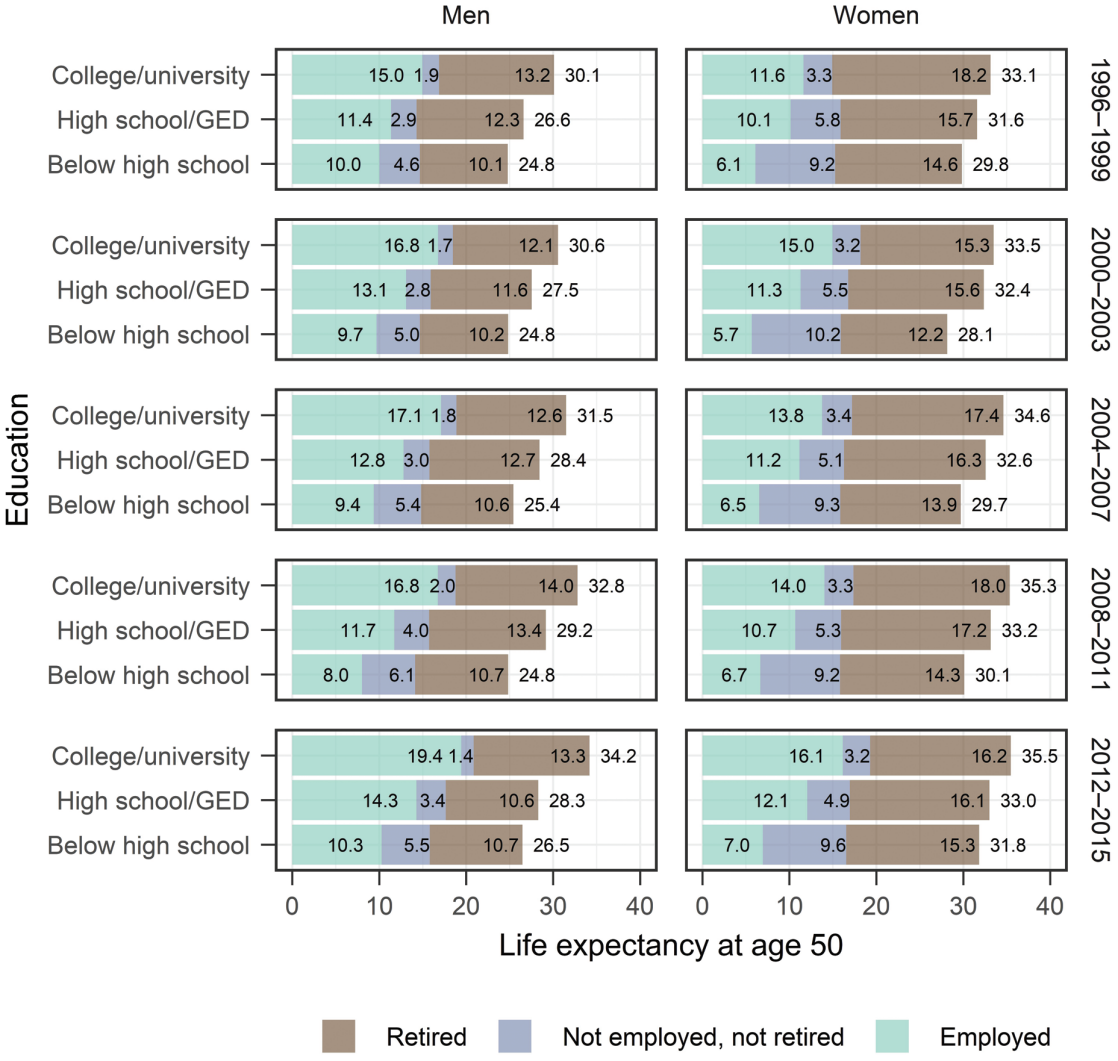
Life expectancy (LE) at 50 and the composition by state. Source: Authors’ calculation based on the Health and Retirement Study, 1996–2016.

While Table 1 described only those individuals who survived to retirement, below, we focus on *all individuals* (i.e., including those who died without retiring). Figure 3 shows a clear educational gradient in LE at age 50 for both genders. Higher education is associated with more time both employed and retired. Those with below high school education spent more years not employed and not retired, especially for women.

We also find that women had a higher RE than men. Across education, the absolute difference in RE between women and men was larger among the college/university group; on the other hand, the gender difference in LE was smaller among the college/university group. On average, women with college/university education had a LE that was 2.6 years higher than their male counterparts, whereas they had a 4.0-year higher RE. This demonstrates that our multistate approach captures additional inequalities due to different work-retirement patterns in addition to LE differences.

**Figure 4.**
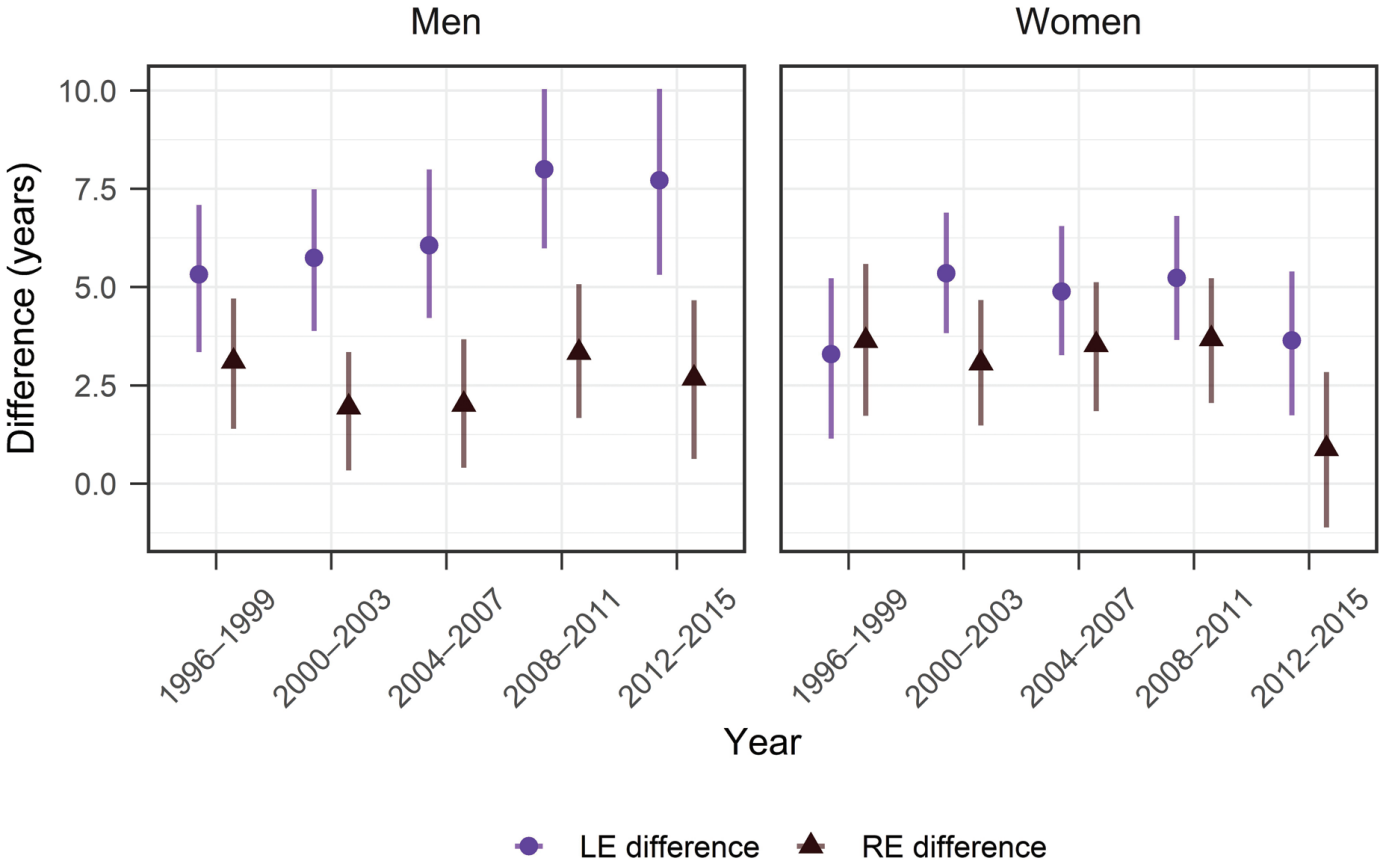
Differences in life expectancy (LE) and retirement expectancy (RE) at age 50 between the lowest and highest education groups. Source: Authors’ calculation based on the Health and Retirement Study, 1996–2016. *Note*: Error bars show 95% bootstrap confidence intervals.

Education is positively associated with both RE and LE. Figure 4 shows differences in RE and LE between people with the highest and lowest education. Differences in RE between educational groups were smaller than differences in LE, particularly for men. This suggests that work dynamics in old ages compensate for the higher mortality of lower-educated people. Among men, although differences in LE (point estimates) increased over time, differences in RE were relatively stable. Again, this indicates that rising inequalities in LE were driven by rising inequalities in time spent working, not in retirement, once their actual work-retirement transitions were considered. For women, the trends of the two measures, as indicated by the confidence intervals, were both unclear.

**Figure 5.**
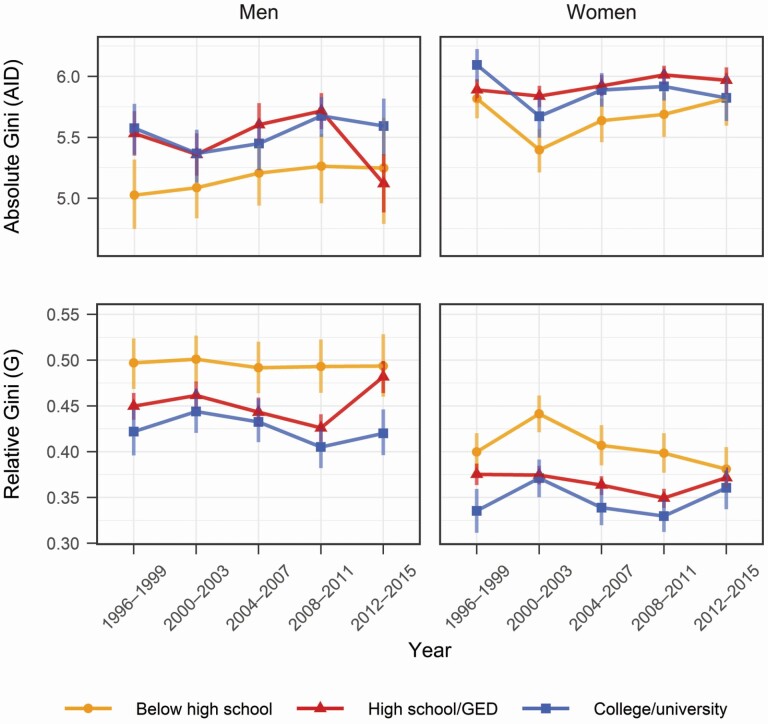
Trends of retirement life-span variation. Source: Authors’ calculation based on the Health and Retirement Study, 1996–2016. *Note*: Calculations are conditional upon surviving to 50, and individuals in all transient states at 50 are included.

Figure 5 shows AID and G of retirement life span by gender and education. Overall, the overlapping confidence intervals indicate that the variation in retirement life span was relatively stable in 1996–2015. The lower-educated had less absolute variation, but given their lower RE, this translates to more relative variation. Similarly, men had lower AID than women, but men had higher G because of their lower RE.

## Discussion

This article examined gender and educational differences in RE and retirement life-span variation in the United States from 1996 to 2015. Despite the longevity improvement at the population level over the study period, we find substantial and consistent inequalities in RE by gender and education. Over the study period, women spent 3.8 years longer in retirement than men, on average. Higher-educated men lived 2.6 years longer in retirement than lower-educated men; the gap for women was 3.0 years. Time spent in retirement varies less within the lower-educated group than it does within the other two groups. However, given their lower RE, this translates into higher relative retirement life-span variation.

### Retirement Expectancy

There is growing interest in tracking trends and disparities in life spans at older ages to better understand the effects of mortality on pension systems as pension reforms are implemented in an aging world (Shi & Kolk, 2022). As a good starting point, researching life spans at pensionable ages (e.g., age 65)—an approach used by most prior studies—facilitates comparisons between income, education, and countries (Chomik & Whitehouse, 2010; Kalwij et al., 2013; Zarulli et al., 2012). Assuming everyone retires at the full retirement age is a useful approach when studying the pension system as a whole on topics such as intergenerational equity and pension sustainability.

Murtin et al. (2022) showed that in 2011 LE at age 65 (LE_65_) was 18.3 years for men and 20.5 years for women. We found that for those who survived to retirement, RE was 15.6 years for men and 18.5 for women in 2008–2011 (Table 1). The discrepancies in LE_65_ and our estimates of RE for retirees were around 2 years, roughly equivalent to the time spent in labor force reentry. Furthermore, as men with higher education spent more time in labor force reentry, the gap in RE between retirees with the lowest and highest education for men (2.5 years) was smaller than the gap in LE_65_ (4.2 years). For women, as time spent in reentry was rather similar across education groups, the gap in RE between retirees with the lowest and highest education (3.4 years) was similar to the gap in LE_65_ (2.8 years). Hence, the conventional approach of using LE_65_ to approximate RE overestimates the actual time in retirement, and may also overestimate the educational gap in RE as individuals with higher education tend to spend more time in reentry.

Additionally, the conventional approach of using LE at age 65 underestimates inequalities in retirement time by ignoring individuals who died without retiring. This makes our study conceptually different from the other studies. Significant numbers of people die before retiring, which partly explains why estimates of LE at “pensionable ages” are higher than our estimates of retirement expectancy at age 50. Men with higher education levels live longer than men with lower educational attainment. The SES-related differences are not as large among women. These key dynamics that are captured in our multistate approach, but not in conventional studies, show a more accurate picture of inequalities in retirement life span.

A few studies have examined RE considering dynamic transitions. For example, Leinonen et al. (2018) used the Sullivan method to compare RE across occupational classes in Finland, and Ghilarducci and Webb (2018) used the nonparametric approach of hot-deck imputation with HRS data to study RE across gender, education, race, etc. They both find that longer RE is associated with higher SES, consistent with our findings. Our patterns of gender and educational differences in the probability of dying without retiring are similar to those found by Ghilarducci and Webb (2018), though we used a period and parametric approach while they used a cohort and nonparametric approach. Our findings are also consistent with the literature on postretirement employment, which highlights the importance of labor force reentry (Cahill et al., 2015). Although men are more likely to reenter the labor force than women (Cahill et al., 2011), interestingly, there are no gender differences in the duration of reemployment (Table 1).

Gender differences in RE were up to 5 years within education groups. This could mainly be explained by women’s lower mortality. One caveat is that we used self-reported information on retirement. Prior research suggests that women’s earlier exits from the labor force due to family caretaking responsibilities make them less likely to identify themselves as retirees (Allmendinger et al., 1992). Gender inequalities in RE would be even larger if providing care for others after leaving the labor force were to be considered retirement. Yet, retiring to do care work is not the same thing as retiring to control the pace and content of one’s time. If caregivers were not perceived as retirees, gender differences in retirement expectations would be smaller.

Lower education is associated with a higher chance of death prior to retirement and lower RE. However, the magnitude of the educational difference is volatile across time, suggesting the important role of external social and economic circumstances, consistent with previous literature on the business cycle and late working life (e.g., Dudel & Myrskylä, 2017). At age 50, more educated individuals not only have a longer working LE but also a longer RE for both men and women. This implies that it might be a high-SES privilege to work longer due to better health.

### Retirement Life Span Variation

An important contribution of our study is that we introduce the concept of (within-group) retirement life-span variation. The AID in retirement life span, interpreted as the average difference in retirement life span between any two random individuals, is 4.8–6.1 years depending on the gender and education group whose retirement expectations ranged between 10.0 and 18.8 years. This suggests substantial within-group heterogeneity in these retirement life spans.

Moreover, to put the gender and educational differences in retirement life-span variation into perspective, the AID and Gini coefficient of remaining life span at age 65 range between 4.5 and 5.0 and between 0.23 and 0.35, respectively, for men in 2015 across all countries in the Human Mortality Database (own calculations; HMD, 2022). Thus, we found that educational differences, as well as gender gaps, in retirement life-span variation in the United States were broadly similar to the male cross-country gap in the overall variation in life spans after age 65, across countries as diverse as Russia, Japan, and the United States. Although the distributions of retirement life spans in this article and life spans after age 65 (the comparison of HMD countries) will differ somewhat, this simple comparison shows that education and gender are stratifying the variation in retirement life spans in the United States to an extent comparable to these large differences in life-span variation across countries.

Gaps in retirement life-span variation between educational groups did not widen over the period, which is in contrast to what has been found for U.S. trends in life-span variation over a broader age range (Sasson, 2016). This might be in part because of the different age ranges examined. A study of long-term trends in life-span variation in Finland showed that the divergence in life-span variation between socioeconomic groups resulted entirely from differential mortality trends at ages below a moving young-old threshold (close to the LE; van Raalte et al., 2014).

The AID and Gini coefficient show different ranks of gender and educational groups. By definition, the Gini coefficient is calculated as AID divided by the mean (in our case, RE), so declines in the Gini coefficient may be driven by increases in the mean (Permanyer & Scholl, 2019). For example, if AID increases from 5 to 6 and RE increases from 10 to 15, correspondingly, the Gini coefficient decreases from 0.5 to 0.4. Hence, the AID and Gini coefficients may show different results. We do not prefer either measure, as they both give complementary perspectives.

### Methodological Considerations

The assumptions on which the models are based need to be considered in interpretation. First, the Markovian assumption, that a transition probability at age *x* only depends on the state at *x* (besides age, period, and education), not prior transition histories, can over-simplify the reality. Among retirees at older ages, people who have unstable employment histories are more likely to die due to possible precarious economic conditions. Taking transition history into account when estimating transition probabilities is unfeasible, as we would need a sufficiently long window of observation and a large sample size, yet such data are rarely available. Second, the multistate life table technique is based on hypothetical cohorts who are assumed to experience stationary transition probabilities. Period changes such as the Covid-19 pandemic that have an impact on the labor market or mortality will affect the experience of the actual retirement life of people. However, these potential challenges do not limit our analysis. For example, the Markov assumption might seem strong, but the most recently occupied state is a very good predictor of transitions, and to some extent, captures the past, as do the socioeconomic variables we use.

To test the robustness of the findings, we used an alternative threshold age of 70 (Figures A2–A4) above which respondents who were in NENR were reclassified as “retired.” The general patterns remain, but the less educated are more affected by the choice of a higher threshold. Consequently, choosing a higher threshold yields larger educational differences, but the changes in magnitude are small.

One important extension of our work would be to include race/ethnicity in the analysis. If we further break down our analysis of the HRS data by race/ethnicity, some groups will have very small sample sizes. Nevertheless, racial/ethnic disparities in mortality and labor participation are important to understand inequalities in the United States, and future work should explore these aspects.

### Policy Implications

Understanding the distribution of retirement life span is important for welfare policies. Providing resources to protect individuals against contingencies, including old age and inability to work is on the global policy agenda. Individuals with poorer health are more likely to quit jobs earlier and less likely to return to work, and they also depend more heavily on welfare programs. Thus, shortfalls in health and economic resources are reflected in retirement, particularly for less-educated individuals and women. Policymakers who aim at equity in social provision for older adults can be better informed by monitoring how these provisions vary across and within gender and education groups. In the United States, social insurance programs such as Social Security and Medicare are based on lifetime work history. The Supplemental Security Income and Medicaid make up some of the differences between individuals with different earnings trajectories. But still, economic security varies substantially among retirees. Higher-earning individuals will have higher retirement benefits (in addition to their private savings), which will widen the well-being gaps between social groups beyond the gaps in LE and RE. A policy-relevant analysis of disparities in expected retirement income over the retirement period would complement our study of RE.

As the population ages, policymakers are concerned about sustainable health care and social security policies. Many countries are encouraging individuals to postpone their retirement (OECD, 2019). It is true that policy discussions to delay the full retirement age for Social Security keep the option of early retirement, such that individuals who enter early retirement due to health and occupational factors can have access to retirement benefits. However, their retirement income streams are reduced due to actuarial adjustments. Thus, raising the full retirement age will inevitably increase the risk of old-age poverty. Future research should incorporate economic security in the analysis of retirement inequalities.

Inequalities in LE have gained increasing attention among researchers who study the fairness and sustainability of health care and pension systems (Auerbach et al., 2017; Goldman & Orszag, 2014). We argue that RE are equally, if not more important, to monitor in this regard. How health, economic, and social factors related to changing inequalities in retirement life span are critical open questions. These factors need to be better understood by actuaries when adjusting forecasts, and by policymakers in changing social insurance or tax policy.

## Supplementary Material

gbac180_suppl_Supplementary_Material

## Author Notes

1. We did not further divide the last category (as some other studies may have done) due to the small samples of earlier birth cohorts that we study.

2. In 1996–2015, the average percentage of employed individuals at age 50 was 80.1% for men and 70.8% for women.

3. See Tables A1–A5 in the Online Appendix for results with 95% bootstrap confidence intervals.
