# Supplementary material for: Inequalities in Retirement Life Span in the United States
Source: J Gerontol B Psychol Sci Soc Sci. 2022 Nov 17;78(5):891–901. doi: 10.1093/geronb/gbac180 (PMC10174282; doi:10.1093/geronb/gbac180)
Supplement: gbac180_suppl_Supplementary_Material [file gbac180_suppl_supplementary_material.pdf]

Supplementary Materials

Table A1. Initial retirement age, with 95% bootstrap confidence intervals.

	Men					Women				
	1996–1999	2000–2003	2004–2007	2008–2011	2012–2015	1996–1999	2000–2003	2004–2007	2008–2011	2012–2015
**Total**	**63.5**	**64.5**	**64.5**	**64.2**	**65.9**	**63.8**	**64.9**	**64.5**	**64.4**	**65.4**
95% CI lower bound	63.2	64.1	64.1	63.8	65.4	63.5	64.5	64.2	64.0	64.9
95% CI upper bound	63.9	64.8	64.9	64.6	66.3	64.2	65.2	64.8	64.7	65.8
**Below high school**	**63.5**	**63.6**	**63.7**	**63.2**	**64.5**	**63.9**	**64.6**	**64.4**	**64.3**	**65.0**
95% CI lower bound	62.9	62.9	63.0	62.5	63.6	63.4	64.0	63.8	63.8	64.2
95% CI upper bound	64.1	64.2	64.4	64.0	65.5	64.4	65.1	65.0	64.9	65.8
**High school/GED**	**62.7**	**64.0**	**63.9**	**63.8**	**65.4**	**64.0**	**64.8**	**64.4**	**64.1**	**65.0**
95% CI lower bound	62.3	63.6	63.4	63.3	64.8	63.6	64.3	64.0	63.6	64.5
95% CI upper bound	63.2	64.5	64.5	64.3	66.0	64.4	65.2	64.8	64.5	65.6
**College/university**	**64.6**	**65.9**	**66.2**	**66.1**	**67.9**	**63.3**	**66.1**	**65.2**	**65.4**	**67.0**
95% CI lower bound	64.0	65.3	65.5	65.4	67.0	62.6	65.2	64.5	64.7	66.1
95% CI upper bound	65.4	66.6	67.0	66.8	68.7	64.1	66.9	65.9	66.0	67.9

Source: Authors' calculation based on the Health and Retirement Study, 1996–2016.

Table A2. Life expectancy (LE) at initial retirement, with 95% bootstrap confidence intervals.

	Men					Women				
	1996–1999	2000–2003	2004–2007	2008–2011	2012–2015	1996–1999	2000–2003	2004–2007	2008–2011	2012–2015
**Total**	**16.2**	**15.9**	**16.7**	**17.7**	**16.2**	**19.7**	**18.9**	**19.7**	**20.4**	**19.8**
95% CI lower bound	15.9	15.5	16.3	17.3	15.7	19.4	18.5	19.4	20.1	19.4
95% CI upper bound	16.5	16.3	17.1	18.1	16.7	20.0	19.2	20.1	20.8	20.2
**Below high school**	**14.3**	**14.4**	**14.9**	**15.0**	**15.2**	**18.4**	**16.4**	**17.9**	**18.3**	**19.2**
95% CI lower bound	13.4	13.6	14.0	13.9	13.8	17.5	15.5	16.9	17.4	18.0
95% CI upper bound	15.5	15.4	15.9	16.1	16.8	19.3	17.2	18.8	19.2	20.5
**High school/GED**	**16.3**	**15.8**	**16.8**	**17.7**	**15.2**	**19.5**	**19.4**	**20.1**	**20.9**	**20.0**
95% CI lower bound	15.5	15.2	16.1	16.9	14.3	18.8	18.9	19.5	20.3	19.3
95% CI upper bound	17.1	16.4	17.6	18.4	16.0	20.1	20.0	20.7	21.6	20.7
**College/university**	**17.1**	**16.4**	**16.9**	**18.4**	**17.7**	**21.3**	**18.9**	**20.8**	**21.4**	**19.9**
95% CI lower bound	16.1	15.4	15.9	17.5	16.6	20.0	17.8	19.7	20.3	18.6
95% CI upper bound	18.2	17.3	17.9	19.3	18.9	22.7	20.0	21.9	22.5	21.2

Source: Authors' calculation based on the Health and Retirement Study, 1996–2016.

Table A3. Retirement expectancy at initial retirement, with 95% bootstrap confidence intervals.

	Men					Women				
	**1996–1999**	**2000–2003**	**2004–2007**	**2008–2011**	**2012–2015**	**1996–1999**	**2000–2003**	**2004–2007**	**2008–2011**	**2012–2015**
**Total**	**14.4**	**13.9**	**14.6**	**15.6**	**14.0**	**17.7**	**16.8**	**17.7**	**18.4**	**17.7**
95% CI lower bound	14.1	13.6	14.3	15.2	13.5	17.5	16.5	17.4	18.1	17.3
95% CI upper bound	14.7	14.2	15.0	15.9	14.5	18.0	17.1	18.0	18.7	18.1
**Below high school**	**12.7**	**12.8**	**13.3**	**13.5**	**13.4**	**16.6**	**14.6**	**15.9**	**16.3**	**17.2**
95% CI lower bound	11.9	12.1	12.4	12.5	12.0	15.8	13.8	15.1	15.4	16.0
95% CI upper bound	13.6	13.7	14.2	14.4	15.0	17.5	15.3	16.8	17.2	18.5
**High school/GED**	**14.5**	**13.8**	**14.8**	**15.5**	**12.9**	**17.4**	**17.2**	**17.9**	**18.8**	**17.8**
95% CI lower bound	13.8	13.2	14.1	14.8	12.1	16.7	16.7	17.3	18.2	17.1
95% CI upper bound	15.2	14.4	15.5	16.2	13.7	18.0	17.8	18.5	19.4	18.5
**College/university**	**15.0**	**14.1**	**14.5**	**15.8**	**15.1**	**19.5**	**16.7**	**18.7**	**19.2**	**17.6**
95% CI lower bound	13.9	13.1	13.5	15.0	14.0	18.2	15.6	17.6	18.2	16.3
95% CI upper bound	16.0	15.0	15.5	16.7	16.2	20.9	17.9	19.8	20.4	18.8

Source: Authors' calculation based on the Health and Retirement Study, 1996–2016.

Table A4. Expected years in re-employment at initial retirement, with 95% bootstrap confidence intervals.

	Men					Women				
	**1996–1999**	**2000–2003**	**2004–2007**	**2008–2011**	**2012–2015**	**1996–1999**	**2000–2003**	**2004–2007**	**2008–2011**	**2012–2015**
**Total**	**1.8**	**2.0**	**2.1**	**2.1**	**2.2**	**2.0**	**2.1**	**2.0**	**2.0**	**2.1**
95% CI lower bound	1.7	1.9	1.9	2.0	2.1	1.8	1.9	1.9	1.9	1.9
95% CI upper bound	2.0	2.2	2.2	2.3	2.4	2.1	2.2	2.2	2.2	2.3
**Below high school**	**1.6**	**1.6**	**1.7**	**1.6**	**1.8**	**1.8**	**1.8**	**1.9**	**2.0**	**2.0**
95% CI lower bound	1.3	1.4	1.4	1.3	1.5	1.6	1.6	1.6	1.7	1.7
95% CI upper bound	2.1	1.8	1.9	1.8	2.1	2.0	2.0	2.2	2.2	2.3
**High school/GED**	**1.8**	**2.0**	**2.0**	**2.2**	**2.2**	**2.1**	**2.2**	**2.2**	**2.2**	**2.2**
95% CI lower bound	1.6	1.8	1.8	2.0	2.0	1.9	2.0	2.0	2.0	2.0
95% CI upper bound	2.1	2.2	2.3	2.4	2.5	2.3	2.4	2.4	2.4	2.4
**College/university**	**2.1**	**2.3**	**2.4**	**2.6**	**2.6**	**1.8**	**2.1**	**2.1**	**2.2**	**2.3**
95% CI lower bound	1.9	2.1	2.1	2.3	2.4	1.6	1.9	1.9	2.0	2.1
95% CI upper bound	2.4	2.6	2.7	2.8	3.0	2.0	2.4	2.4	2.4	2.6

Source: Authors' calculation based on the Health and Retirement Study, 1996–2016.

Table A5. Life expectancy (LE) % in re-employment at initial retirement, with 95% bootstrap confidence intervals.

	Men					Women				
	1996–1999	2000–2003	2004–2007	2008–2011	2012–2015	1996–1999	2000–2003	2004–2007	2008–2011	2012–2015
**Total**	**11.2**	**12.6**	**12.3**	**12.1**	**13.7**	**9.9**	**11.0**	**10.2**	**9.9**	**10.6**
95% CI lower bound	10.3	11.8	11.4	11.2	12.8	9.3	10.4	9.6	9.3	9.9
95% CI upper bound	12.1	13.5	13.3	13.0	14.9	10.6	11.7	11.0	10.6	11.4
**Below high school**	**11.1**	**11.1**	**11.1**	**10.4**	**11.9**	**9.7**	**11.0**	**10.8**	**10.7**	**10.4**
95% CI lower bound	9.5	9.7	9.5	8.8	10.1	8.5	9.7	9.4	9.4	9.0
95% CI upper bound	13.7	12.4	12.6	11.9	13.9	11.0	12.3	12.2	12.0	11.8
**High school/GED**	**10.9**	**12.7**	**12.1**	**12.3**	**14.8**	**10.8**	**11.3**	**10.8**	**10.4**	**11.1**
95% CI lower bound	9.9	11.6	11.1	11.3	13.5	9.9	10.5	10.0	9.6	10.2
95% CI upper bound	12.3	13.8	13.4	13.5	16.1	11.7	12.1	11.8	11.2	12.0
**College/university**	**12.4**	**14.2**	**14.2**	**13.9**	**14.9**	**8.4**	**11.4**	**10.2**	**10.2**	**11.8**
95% CI lower bound	10.9	12.8	12.7	12.6	13.3	7.5	10.1	9.1	9.1	10.4
95% CI upper bound	14.0	15.7	16.0	15.5	16.8	9.5	12.8	11.4	11.3	13.2

Source: Authors' calculation based on the Health and Retirement Study, 1996–2016.

Figure A1. Trends of retirement expectancy, with 95% bootstrap confidence intervals.

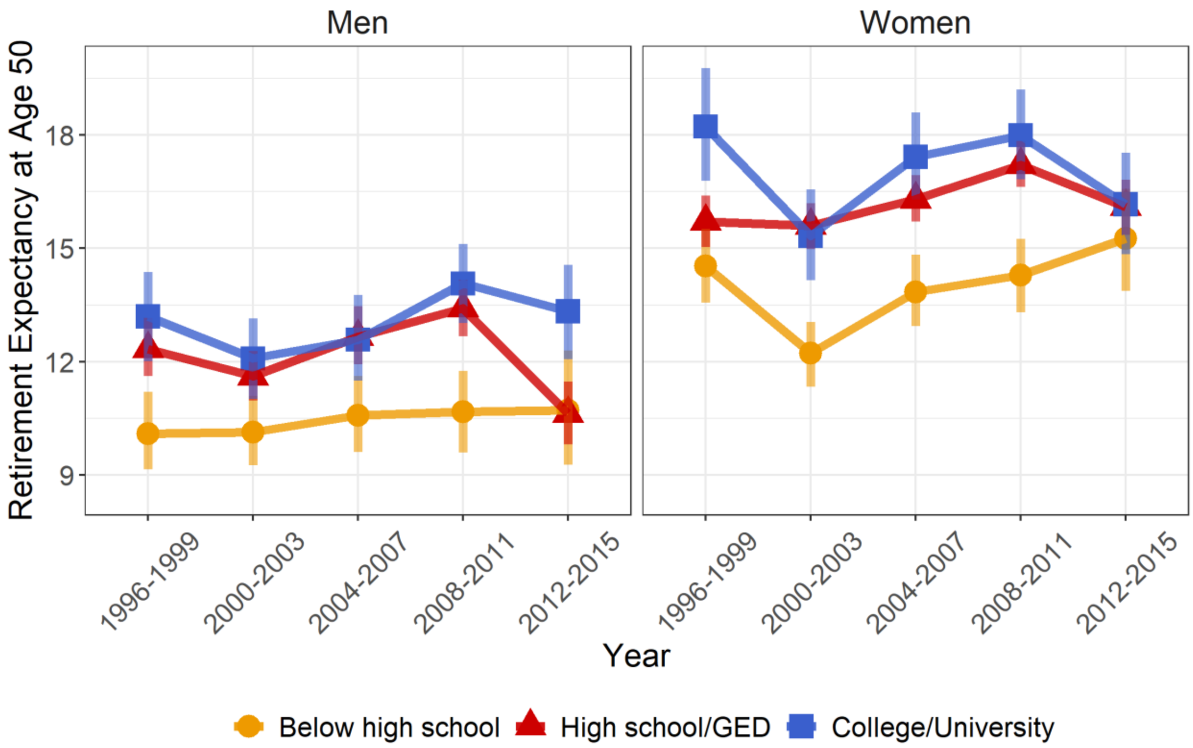

Source: Authors' calculation based on the Health and Retirement Study, 1996–2016. *Note:* Calculations are conditional upon surviving to 50, and individuals in all transient states at 50 are included.

Figure A2. Percentage of individuals not surviving to retirement, with retirement threshold age 70.

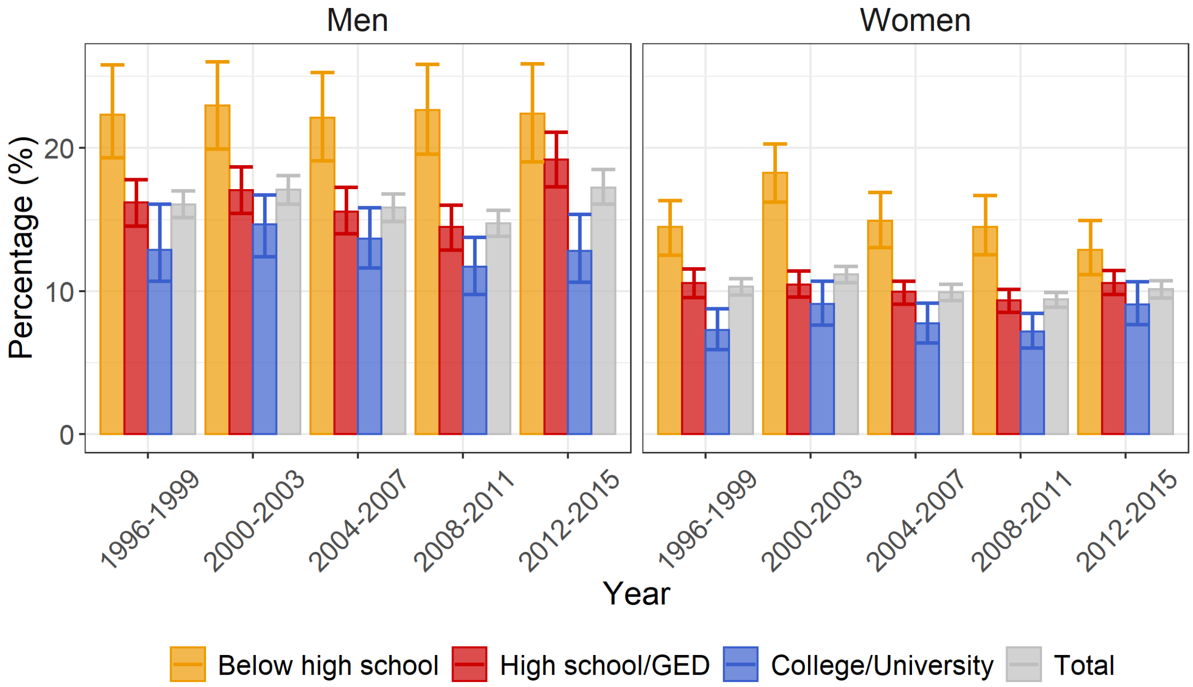

Source: Authors' calculation based on the Health and Retirement Study, 1996–2016. *Note:* Error bars show 95% bootstrap confidence intervals.

Figure A3. Trends of retirement expectancy, with retirement threshold age 70.

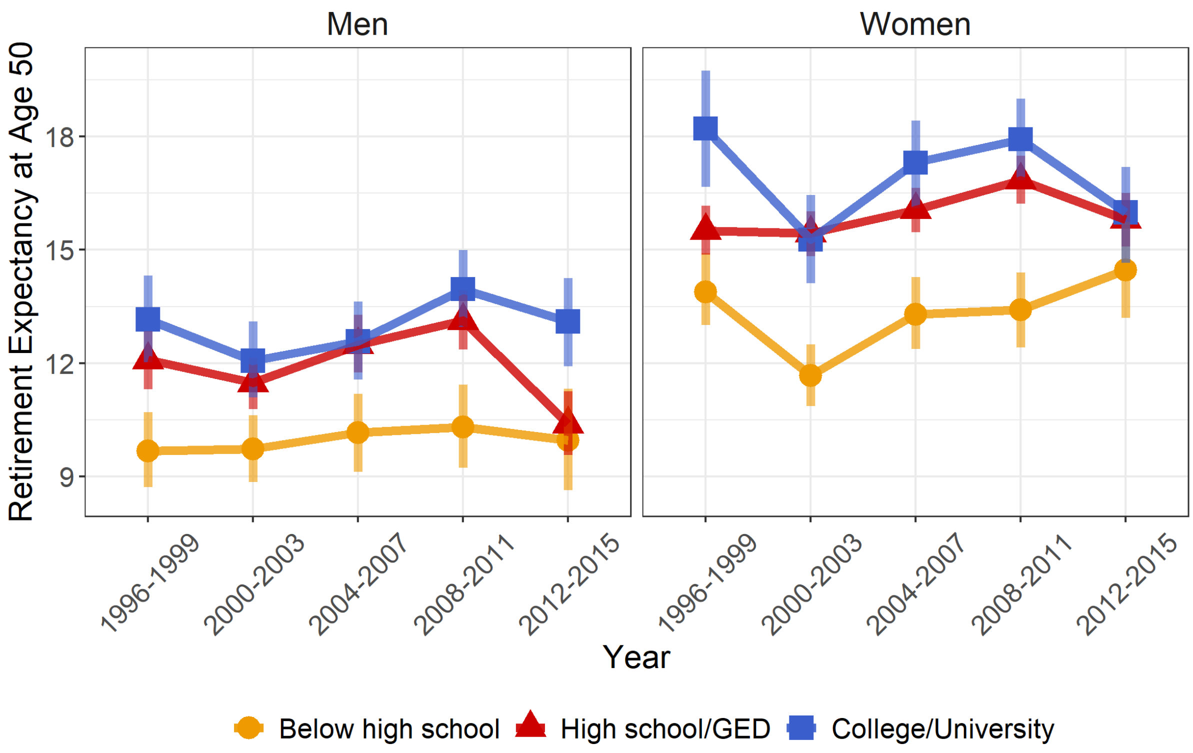

Source: Authors' calculation based on the Health and Retirement Study, 1996–2016. *Note:* Calculations are conditional upon surviving to 50, and individuals in all transient states at 50 are included.

Figure A4. Trends of retirement lifespan variation, with retirement threshold age 70.

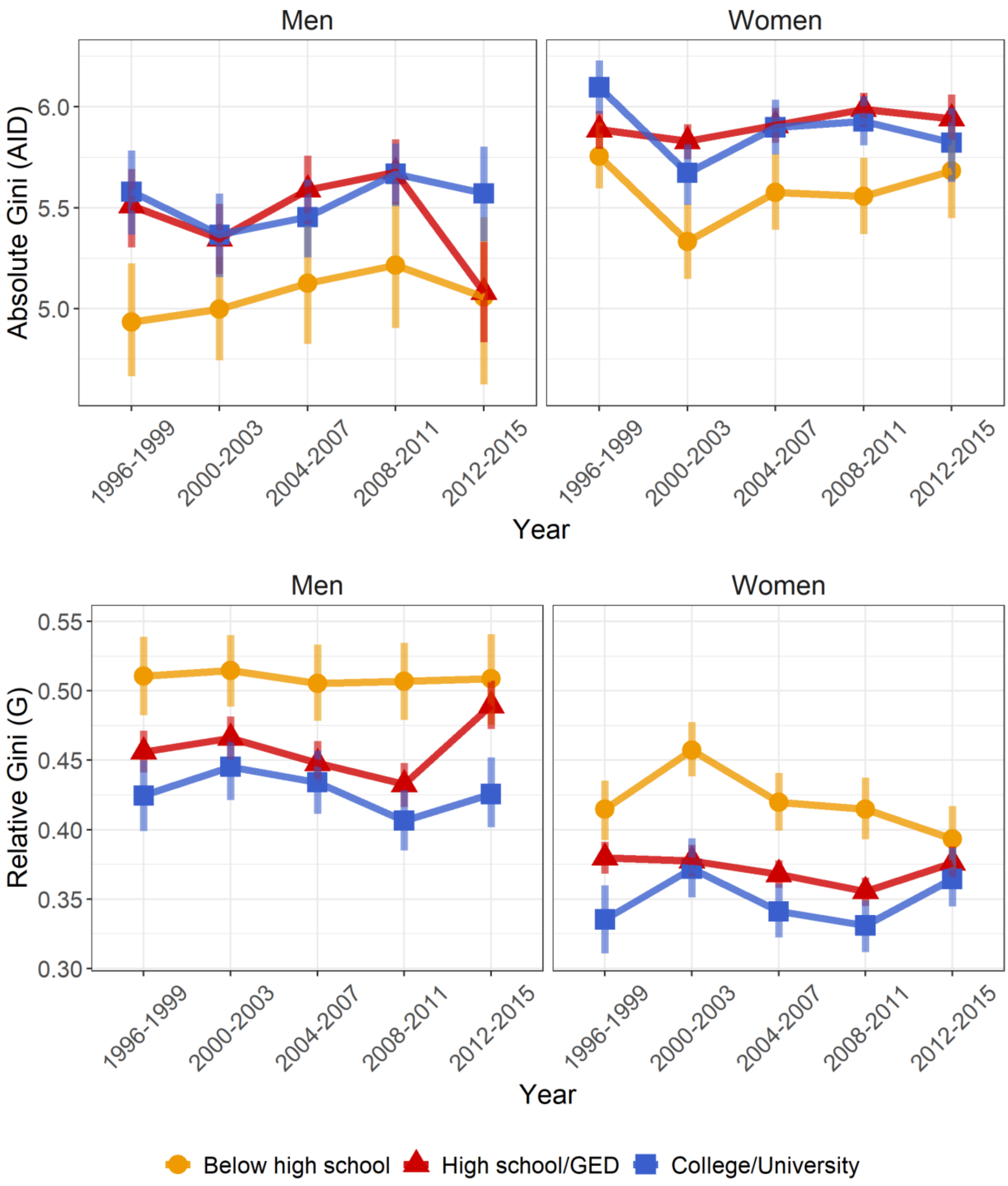

Source: Authors' calculation based on the Health and Retirement Study, 1996–2016. *Note:* Calculations are conditional upon surviving to 50, and individuals in all transient states at 50 are included.
